# Test-Retest Reliability and Validity of TecnoBody D-Wall to Assess the Range of Motion During Overhead Squat in Healthy Individuals

**DOI:** 10.3390/life15010080

**Published:** 2025-01-10

**Authors:** Caglar Soylu, Gorkem Acar, Berkay Uzumcu, Pervin Demir, Sinan Seyhan, Turker Biyikli

**Affiliations:** 1 Gulhane Faculty of Physiotherapy and Rehabilitation, University of Health Sciences, 06010 Ankara, Turkey; 2 Department of Sport Science, Institute of Graduate Education, Manisa Celal Bayar University, 45040 Manisa, Turkey; gorkemacar2@gmail.com; 3 Berton Robotic Technology and Health Inc., 34720 Istanbul, Turkey; berkayuzumcu59@gmail.com; 4 Biostatistics and Medical Informatics, Basic Medical Sciences, Faculty of Medicine, Ankara Yildirim Beyazit University, 06145 Ankara, Turkey; pervin.demr@gmail.com; 5 Department of Coaching Education, Faculty of Sport Sciences, Manisa Celal Bayar University, 45040 Manisa, Turkey; sinan.seyhan@cbu.edu.tr; 6 Department of Coaching Education, Marmara University Faculty of Sport Sciences, 34815 Istanbul, Turkey; turker.biyikli@marmara.edu.tr

**Keywords:** movement biomechanics, TecnoBody, test-retest reliability, functional movement analysis

## Abstract

This study evaluated the validity and reliability of the TecnoBody D-Wall system in assessing joint range of motion (ROM) during overhead squat movements in healthy individuals, using Kinovea as a reference tool for data comparison. A total of 29 participants (16 males, 13 females) with a mean age of 28.41 ± 6.66 years were included. Measurements were conducted for hip and knee joint angles in the sagittal plane, with three repetitions per participant analyzed using both systems. The D-Wall system employed a 3D Kinect V2 camera and force platform, while Kinovea used 2D video-based motion analysis. The results demonstrated excellent agreement between the two systems, with intra-class correlation coefficients (ICC) ranging from 0.79 to 0.99. For the knee joint, the test-retest ICC values were 0.99 (95% CI: 0.97–0.99) for Kinovea and 0.98 (95% CI: 0.95–0.99) for the D-Wall on the right side, and 0.98 (95% CI: 0.97–0.99) for Kinovea and 0.88 (95% CI: 0.79–0.94) for the D-Wall on the left side. For the hip joint, test-retest ICC values were 0.99 (95% CI: 0.97–0.99) for Kinovea and 0.94 (95% CI: 0.88–0.97) for the D-Wall on the right side, and 0.98 (95% CI: 0.97–0.99) for Kinovea and 0.93 (95% CI: 0.87–0.97) for the D-Wall on the left side. Bland–Altman plots confirmed good agreement, with no significant systematic bias observed. Both systems showed statistically insignificant differences (*p* > 0.05) between measurements, and correlation values ranged from 0.83 to 0.99, indicating strong associations. These findings highlight the high validity and reliability of both TecnoBody D-Wall and Kinovea systems in measuring joint angles during dynamic movements. The comparable accuracy between systems suggests that either system can be effectively utilized in clinical or research settings, depending on specific needs and resource availability.

## 1. Introduction

The measurement of joint range of motion (ROM) is a critical component in evaluating human movements, encompassing both static and dynamic, passive and active motions, and providing essential insights into joint functionality or dysfunction. While the importance of ROM assessment has long been recognized, advances in measurement tools have significantly enhanced precision and accessibility compared to traditional methods. Accurate ROM assessment remains integral to musculoskeletal evaluations and rehabilitation planning, forming a fundamental aspect of clinical practice for healthcare professionals such as doctors and physiotherapists [1]. Traditionally, hand-held goniometers have been considered the gold standard for measuring joint ROM due to their objectivity and reliability, although they present challenges, such as stabilizing the reference arm during joint rotation, which may limit their practicality in certain scenarios [2,3].

However, the use of hand-held goniometers presents notable limitations, particularly when measuring actively assisted ROM. These challenges include difficulty in stabilizing the device’s reference arm during movement, inaccuracies in interpreting angles at the limits of ROM, and unintended joint movements when detaching the goniometer, potentially introducing errors in data collection. In this context, digital inclinometers offer significant advantages, such as their ability to reference fixed angles, provide easily readable digital displays, and secure precise measurements without inducing extraneous movement [1,2,3]. Despite these potential benefits, the reliability of ROM measurements obtained via digital inclinometers remains underexplored in the literature [4]. Importantly, the reliability of any measurement device is a fundamental consideration in both clinical and research settings.

The D-Wall device (TecnoBody SRL, Bergamo, Italy) represents a cutting-edge evaluation and rehabilitation tool, designed to enhance movement quality through real-time auditory and visual feedback during rehabilitation and adapted physical activities, with its primary applications in sports sciences. The device facilitates comprehensive assessments of mobility, aerobic performance, postural alignment, segmental and global coordination, and sensorimotor skills. Furthermore, it aids in addressing joint asymmetries, analyzing movement kinematics, and evaluating biomotor abilities such as agility and balance across key anatomical regions, including the head, trunk, shoulders, hips, and knees. Equipped with a 3D camera and a force platform, the D-Wall captures real-time movements of up to 16 body joints, offering immediate biofeedback. This capability not only enables objective evaluation but also allows for tailored program development, such as rehabilitation protocols, with pre- and post-assessment comparisons to monitor progress and outcomes [5].

Similarly, Kinovea, an open-source software (2023.1.2) released under a GPLv2 license in 2009, is widely utilized for two-dimensional kinematic analysis using videos captured with standard cameras. Kinovea offers a range of kinematic parameters and has found applications in sports science, clinical analyses, and comparative validation of emerging technologies. Its accessibility, requiring only a basic camera and computer setup, and its markerless tracking capabilities make it a versatile and cost-effective tool for clinical and research purposes. Previous studies have established the validity and reliability of Kinovea in motion analysis, with foundational research published in 2012 and subsequent studies in 2015 and 2020 further reinforcing its utility [6,7,8,9,10,11,12].

Despite the widespread use of the D-Wall device, there is a paucity of research directly comparing its performance against other validated methods like Kinovea. This study aims to address this gap by evaluating the validity and reliability of ROM measurements obtained with the D-Wall device, specifically during overhead squat movements in healthy individuals. The authors hypothesize that both the D-Wall system and Kinovea software will exhibit high test-retest reliability in assessing ROM. However, due to differences in technology and methodology, it is anticipated that the D-Wall system, being more advanced, will demonstrate greater consistency and precision compared to Kinovea, which relies on simpler digital tools. The findings of this study are expected to provide valuable insights into the practical applications and limitations of these systems in clinical and sports science contexts.

## 2. Materials and Methods

### 2.1. Participants

The study was conducted at the Cyber Robotic Center, located in Istanbul, Turkey, between February 2024 and June 2024. This study approved by the Ethics Committee of University of Health Sciences (approval number: 2024/345) and adhered strictly to the principles outlined in the Declaration of Helsinki. The participants were fully informed about the study’s purpose, procedures, potential risks, and benefits. Written informed consent was obtained from all participants before their involvement, ensuring compliance with ethical standards and safeguarding participant autonomy, confidentiality, and rights throughout the research process. A total of 29 healthy individuals, comprising 16 males and 13 females, were recruited for the study. The participants had an average age of 28.41 ± 6.66 years and an average weight of 75.14 ± 24.97 kg. The recruitment criteria were meticulously designed to align with established protocols in movement analysis research, emphasizing the importance of healthy, non-impaired subjects for obtaining reliable and valid ROM measurements [13]. The inclusion criteria required participants to be aged between 18 and 65 years and to voluntarily agree to participate in the study. To minimize confounding factors, the exclusion criteria eliminated individuals with any of the following conditions:

- Lower extremity surgery within the last six months.
- Evidence of pronation in the subtalar angle during exertion.
- Knee conditions that precluded squatting.
- A body mass index (BMI) greater than 30.
- Orthopedic foot deformities or any other conditions that restricted normal joint movement.

The sample size was determined based on a pilot study that produced an intraclass correlation coefficient (ICC) range of 0.779–0.993. To achieve 90% statistical power with a 0.05 type I error rate, the minimum required sample size for an ICC value of 0.90 or higher between two measurements was calculated to be 25 participants. To account for potential dropouts (~10%), the final sample size was increased to 29 participants. This approach aligns with guidelines for sample size estimation in reliability research [14,15], ensuring the statistical robustness of the study.

### 2.2. Experimental Design and Procedures

The participants completed three repetitions of the overhead squat, which is a widely utilized movement in biomechanical assessments due to its ability to evaluate lower extremity joint mechanics. To ensure accurate visual tracking of anatomical markers, the participants were instructed to wear tight-fitting, non-reflective clothing and avoid black or white garments that could interfere with marker detection. Each participant underwent a structured warm-up session and practice trials before formal testing to familiarize themselves with the procedure and reduce movement variability. The warm-up protocol consisted of exactly five minutes of cycling at a moderate intensity (50–60% of maximum heart rate), followed by three sets of 10 dynamic stretches, including forward lunges, leg swings, and bodyweight squats, specifically targeting the hip and knee joints. The testing environment was carefully controlled to enhance measurement reliability. The squats were performed in a well-lit room with uniform ambient lighting, ensuring optimal visibility of anatomical markers. The testing area was equipped with a stable, non-slippery surface to ensure participant safety and consistency during movements.

### 2.3. Measurement Systems

The study employed two complementary systems for ROM analysis: the TecnoBody D-Wall system (TecnoBody, Bergamo, Italy) and the Kinovea software (2023.1.2). The Kinovea software (open-source, developed by Joan Charmant, France, available at www.kinovea.org) (accessed on 21 February 2024) is a 2D video-based motion analysis tool. These tools provided comprehensive kinematic data, combining 3D and 2D motion analysis to assess joint angles at the hip, knee, and ankle in the sagittal plane.

**Figure 1:**
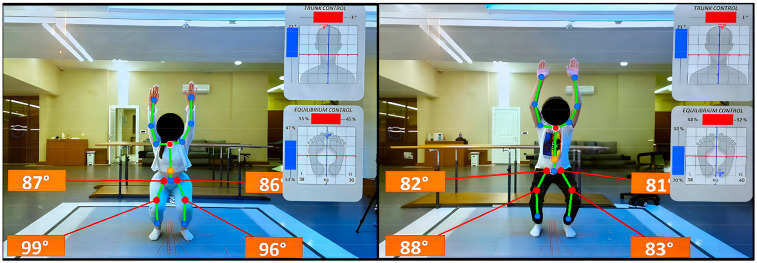
Overhead squat evaluation with TecnoBody D-Wall.

The D-Wall system integrated advanced technologies to capture biomechanical data in real time. It utilized a Full-HD 3D Kinect V2 camera operating at 30 frames per second, positioned 97 cm above ground level. The embedded force platform measured vertical load parameters, allowing for detailed analysis of movement dynamics. Joint angles were recorded with a resolution of 0.1 degrees at a sampling rate of 100 Hz, enabling precise tracking of joint mechanics. The device also provided real-time auditory and visual biofeedback to participants, facilitating improved movement quality during assessments. The D-Wall’s physical dimensions included a wall area of 2.4 m × 2.5 m × 0.18 m and a floor area of 0.03 m × 2.5 m × 3.5 m. These features allowed the device to simultaneously analyze up to 16 body joints, making it a versatile tool for functional movement evaluation (Figure 1).

**Figure 2:**
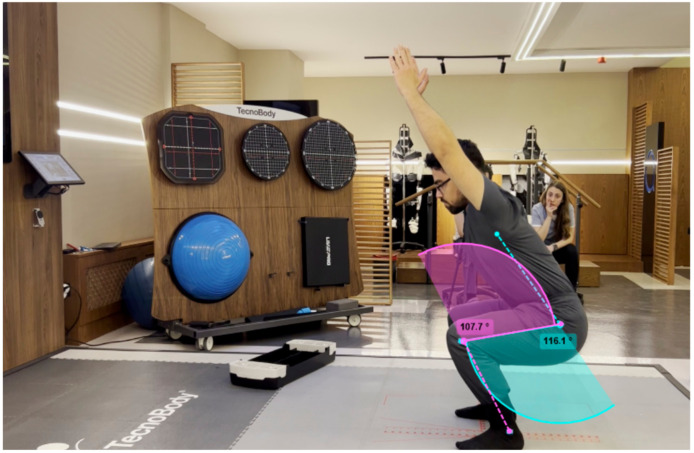
Range of motion measurement with Kinovea program. The purple color represents the hip flexion joint angle, while the green color represents the knee flexion joint angle.

The Kinovea software, an open-source 2D kinematic analysis tool, was used to complement the D-Wall system. High-resolution cameras operating at 60 frames per second were positioned at a perpendicular angle (90°) to the participant. One camera was placed on the right side and another on the left side of the participant, each positioned at a distance of 2 m and a height of 97 cm. Anatomical markers were carefully placed at the anterior superior iliac spine, lateral joint line of the knee, and lateral malleolus, following established guidelines to ensure consistent placement. These markers facilitated accurate measurement of joint angles in the sagittal plane. Kinovea’s built-in angle measurement tools were used to calculate joint ROM, providing a reliable dataset for comparison with the D-Wall system [13,16,17,18] (Figure 2).

### 2.4. Data Collection

Data collection involved three repetitions of the overhead squat for each participant, with movements analyzed in the sagittal plane. Both the D-Wall and Kinovea systems were utilized to provide complementary kinematic data. The D-Wall system recorded joint angles with high precision, leveraging its 3D Kinect V2 camera and force platform to capture vertical load parameters alongside joint mechanics. The system’s high sampling rate and resolution ensured that even subtle variations in joint angles were accurately recorded. Kinovea supplemented these data with 2D video-based motion analysis, using its high-resolution cameras to capture joint movements at a frame rate of 60 fps (frames per second). To ensure consistency and accuracy, the participants were introduced to the testing procedures through warm-up exercises and practice trials. These preparatory steps minimized variability in performance and enhanced familiarity with the testing environment. Four range of motion (ROM) measurements were recorded for each participant, specifically focusing on hip and knee flexion on both the right and left sides. The mean ROM value from the three repetitions was selected for the final analysis to ensure consistency and reliability in the data. This approach aligns with established protocols in movement science, where averaging repeated measurements is considered a robust method for assessing validity and reliability. The physical specifications of the D-Wall, combined with its real-time feedback mechanisms, provided a detailed analysis of joint mechanics during the overhead squat. Similarly, Kinovea’s precise 2D kinematic analysis capabilities ensured a robust dataset for cross-validation of results.

### 2.5. Statistical Analysis

The normal distribution of data was analyzed using the Shapiro–Wilk test and visually examined through histogram graphs. The mean (95% CI lower-upper bound) was provided for the summary of numerical variables. The concordance between the three measurements obtained through Kinovea and D-Wall was evaluated using the ICC (mean measure, two-way mixed-effects model where the participants’ effects are random and the measures’ effects are fixed, with absolute agreement). The values for validity and reliability using Kinovea and D-Wall were calculated based on the average of these three measurements. Test-retest reliability was assessed using two separate measurement sessions conducted 7days apart, performed by the same rater at the same time of day to minimize variability. All evaluations were conducted under similar environmental conditions, with a temperature of approximately 21 °C and humidity of around 60% [13]. The presence of a systematic difference between the two methods (Kinovea and D-Wall) was examined using the Paired *t*-test. Additionally, the agreement between the two sessions for test-retest reliability was analyzed using the ICC (single measure, two-way mixed-effects model where participants’ effects are random and measures’ effects are fixed, with absolute agreement). An ICC value > 0.76 was considered good reliability, while >0.90 was considered excellent reliability [19]. The presence of systematic bias between the two sessions was further examined using the Paired Sample *t*-test. The Spearman rank correlation coefficient was utilized to investigate the relationship between the measurement results obtained from the two methods (Kinovea and D-Wall). While the data were numerical and normally distributed, the Spearman test was chosen to evaluate monotonic relationships without assuming linearity, which is a key advantage when comparing data from different measurement systems. The paired *t*-test, a parametric test, was employed to assess systematic differences between the mean values of the measurements obtained during test-retest reliability and between the two methods. This test was selected because the normality of the data was confirmed using the Shapiro–Wilk test, making the *t*-test appropriate for detecting mean differences under normally distributed conditions. Using both tests allowed us to comprehensively analyze the relationships (Spearman test) and differences (*t*-test) between the measurement systems and sessions, leveraging the strengths of each statistical approach to provide robust results. For evaluating reproducibility, the Bland–Altman plot was used to provide a visual representation of the agreement between the two measurements. The center line on the plot represents the mean difference between the two measurements, and the upper and lower limits of agreement were calculated as the mean difference ±1.96 standard deviation of the differences. If the differences between the two measurements were within acceptable limits and showed consistency, it suggested good reproducibility. All statistical analyses and calculations were conducted using the free R programming language. The utilized packages were as follows: “rel”, “ggplot2”, and “blandr”. A significance level of 0.05 was accepted for all analyses.

## 3. Results

**Figure 3:**
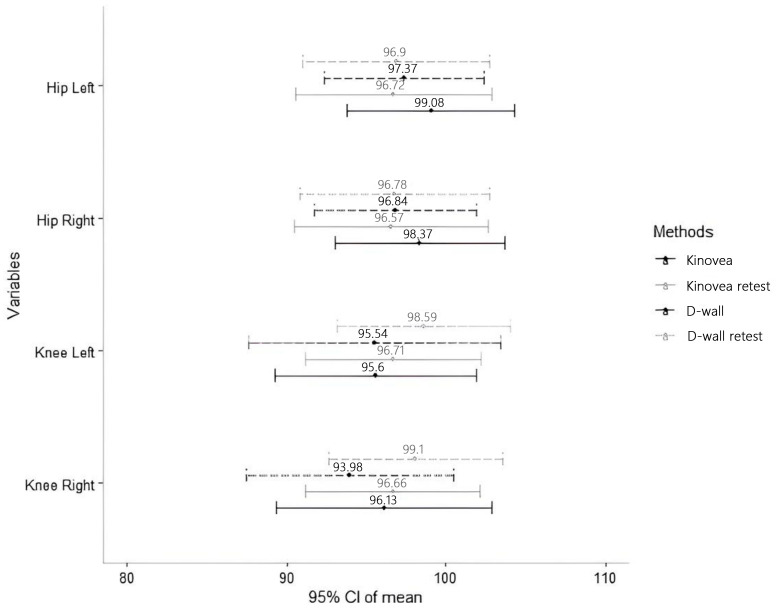
The mean and 95% confidence interval of the measurements obtained from Kinovea, D-wall, and retest evaluation. Comparison of the 95% confidence intervals (CI) for mean joint range of motion (ROM) measurements across different methods (Kinovea and D-Wall) and sessions (test and retest) for the left and right hip and knee joints. The solid black lines (—) represent Kinovea test measurements, the dashed gray lines (--) represent Kinovea retest measurements, the solid dashed black lines (— --) represent D-Wall test measurements, and the dashed gray lines (--) represent D-Wall retest measurements. The overlapping confidence intervals indicate the agreement between methods and sessions.

The agreement in the three measurements of knee and hip for both right and left sides using Kinovea and D-wall was excellent (>0.90, Table 1). The comparison between the two methods and the assessment of test-retest reliability was based on the mean values among these three measurements (Figure 3).

Both the first and second measurements were compared between Kinovea and D-wall. The measurement results from Kinovea and D-wall were similar (*p* > 0.05). The agreement values between the two measurements were high (Table 2). Similarly, the correlation between the obtained measurements was significant and ranged from 0.83 to 0.99 (*p* < 0.05).

**Figure 4:**
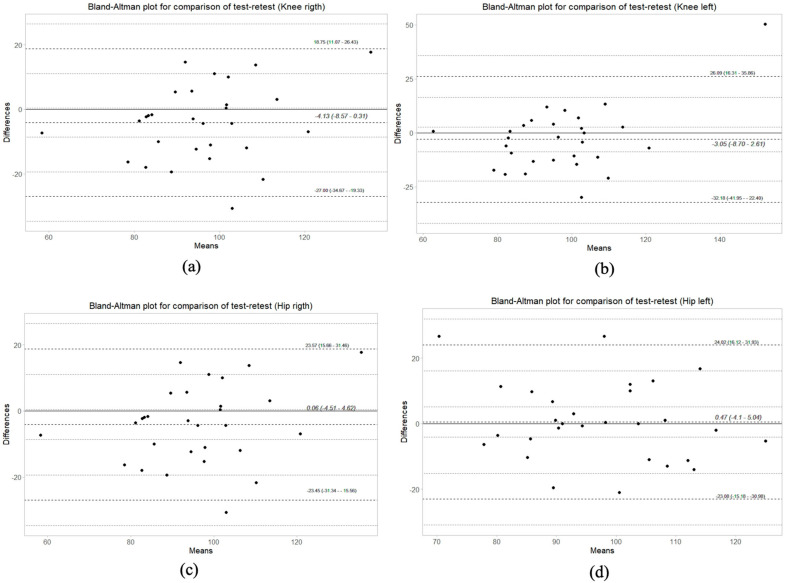
The Bland–Altman plots for the test–retest reliability indicate that (**a**,**b**) represent the D-wall right and left knee measurements, respectively, while (**c**,**d**) represent the D–wall right and left hip measurements. The dashed lines show the limits of agreement and mean of differences, and the dotted lines represent the 95% CI upper and lower limits.).

The errors obtained for Kinovea and D-wall were summarized in Table 3. The ICC values ranged from 0.79 to 0.83, indicating good reliability. No systematic difference was detected among D-wall test-retest measurement results (*p* > 0.05). Upon examining measurement errors, it was determined that the highest error value for D-wall was observed in the Knee Left measurement. To evaluate the reproducibility of D-wall over time, the Bland–Altman plot was used (Figure 4a–d). When the points were evenly distributed above and below the center line and within limits of agreement, was suggested good agreement between the two measurements.

## 4. Discussion

This study evaluated the validity and reliability of the TecnoBody D-Wall and Kinovea systems for measuring joint ROM during functional overhead squats. The findings demonstrated high levels of agreement between the two systems, with excellent ICC values (≥0.90) for hip and knee joints on both sides.

The agreement between Kinovea and D-Wall in ROM measurements reflects their reliability and consistency, as evidenced by the ICC values ranging from 0.79 to 0.99 (Table 1). The Bland–Altman analysis further confirmed these findings, showing no significant systematic bias between test-retest evaluations (Figure 4). Previous studies, such as those by Puig-Diví et al. [18] and Russo et al. [16], also highlighted the importance of precision in digital systems when measuring dynamic movements. These findings suggest that both tools are effective for assessing functional movements, such as squats, in both healthy individuals and potentially in rehabilitation populations.

The high agreement between the TecnoBody D-Wall and Kinovea systems in measuring joint ROM aligns with previous research validating digital motion analysis tools for clinical and sports applications. For instance, Fernández-González et al. [12] demonstrated that Kinovea provided reliable measurements comparable to a 3D motion capture system for gait analysis in healthy participants. Similarly, Puig-Diví et al. [18] highlighted Kinovea’s reliability and validity for capturing kinematic parameters, emphasizing its potential for low-cost and portable motion analysis.

The findings in this study extend the literature by demonstrating excellent reliability (ICC ≥ 0.90) for both systems in assessing dynamic movements like overhead squats. This is consistent with the work of Krause et al. [13], who reported similar levels of reliability using smartphone-based motion analysis tools for assessing lower limb biomechanics. The high ICC values observed in both systems support their utility for ROM assessments in functional movements, reinforcing previous claims that modern digital tools can provide data comparable to traditional gold-standard systems, such as goniometers and optical motion capture systems [20,21].

The slight discrepancies in measurements between the D-Wall and Kinovea systems, particularly for left knee ROM, may reflect differences in technology and methodology. D-Wall’s 3D Kinect V2 camera captures movements at 30 fps with real-time biofeedback, whereas Kinovea relies on 2D video analysis at 60 fps. While Fernández-González et al. [12] and Russo et al. [16] have noted that 3D systems often outperform 2D tools in capturing complex joint kinematics, particularly in dynamic tasks like squats or running; this was not observed in our study. Our findings demonstrated excellent agreement between the two systems for both hip and knee ROM measurements, suggesting that Kinovea’s accessibility and ease of use make it a reliable alternative to 3D systems like D-Wall, particularly in environments where 3D systems are unavailable.

The role of anatomical marker placement is another critical factor influencing the accuracy and reliability of motion analysis systems. Takeda et al. [22] emphasized that consistent and precise marker alignment significantly impacts the validity of ROM measurements. This study adhered to established protocols for marker placement, which likely contributed to the high ICC values observed. Comparable findings have been reported by Sañudo et al. [9], who validated marker-based motion analysis tools for assessing resistance exercises.

Environmental factors, such as lighting and camera positioning, also influence measurement accuracy. Previous studies, including Puig-Diví et al. [18] and Russo et al. [16], highlighted the sensitivity of 2D motion analysis systems to factors such as lighting, camera alignment, and participant positioning. However, in this study, the higher error values observed in left knee measurements during test-retest reliability were associated with the 3D analysis system (D-Wall), rather than the 2D system (Kinovea). This discrepancy may be attributed to several factors inherent to 3D systems. For instance, the Kinect V2 sensor used in the D-Wall system is known to be sensitive to changes in environmental conditions, such as lighting variations and reflective surfaces, which can interfere with depth sensing and joint detection. Furthermore, the reliance of the D-Wall on real-time processing and biofeedback may introduce variability when capturing subtle joint movements, particularly during repetitive tasks. In contrast, the Kinovea system, which relies on 2D video-based motion analysis, appears to be less affected by these specific environmental factors, leading to more consistent measurements. Nevertheless, both systems can benefit from ensuring standardized testing conditions, including consistent lighting, camera alignment, and participant positioning. These measures, as recommended by Ellis et al. [23] and Koo and Li [19], can help mitigate external influences on measurement accuracy. Additionally, regular calibration of 3D sensors and careful attention to placement and alignment during setup may reduce errors and enhance the reliability of 3D systems in dynamic movement analysis.

In addition to technical considerations, this study contributes to the growing body of research on the clinical applicability of motion analysis systems. Mullaney et al. [4] demonstrated the value of digital systems for shoulder ROM assessment in rehabilitation settings, while Bravi et al. [17] validated an instrumented treadmill for gait analysis in neurological rehabilitation. The findings of this study suggest that both the D-Wall and Kinovea systems could be integrated into similar clinical protocols for assessing joint function and tracking rehabilitation progress.

Lastly, the integration of 2D and 3D systems for comprehensive motion analysis has been proposed in several studies. For example, Fernández-González et al. [12] suggested that combining Kinovea’s cost-effective 2D analysis with high-end 3D systems could offer a practical solution for diverse clinical and research needs. This study provides further evidence supporting such an approach, demonstrating that both systems have complementary strengths that can enhance the scope and accuracy of motion analysis.

Despite the promising findings, this study had some limitations. The reliance on consistent marker placement and environmental conditions introduces potential variability in results. Higher error values observed in left knee measurements suggest that additional calibration protocols may be necessary for optimal use of the D-Wall system. Additionally, the study was conducted on healthy individuals, which limits the generalizability of the findings to clinical populations with joint impairments or musculoskeletal disorders. Future studies should explore the applicability of these systems in diverse populations and investigate strategies to integrate 2D and 3D analyses for comprehensive motion assessments.

## 5. Conclusions

This study confirmed the validity and reliability of the TecnoBody D-Wall and Kinovea systems for assessing joint range of motion (ROM) during functional movements such as the overhead squat. Both systems demonstrated excellent agreement, with ICC values exceeding 0.90, establishing their utility in clinical and sports settings. The D-Wall system’s 3D capabilities and real-time biofeedback make it a valuable tool for rehabilitation, allowing clinicians to guide movement patterns effectively, while Kinovea’s cost-effectiveness and portability provide practical solutions for environments with limited access to advanced systems.

Clinically, these findings suggest that the D-Wall and Kinovea systems can support a range of applications, from evaluating ROM in post-surgical patients to tracking athletic performance. The combination of these tools offers opportunities to integrate detailed 3D analysis with accessible 2D methods, optimizing assessments in diverse settings. Future research should focus on expanding these findings to clinical populations, addressing limitations such as environmental consistency and marker placement, and exploring the combined use of these technologies to enhance rehabilitation and performance monitoring.

## Figures and Tables

**Table 1:** The coefficient of agreement among the three measurement results.

	Kinovea		D-Wall	
	Test ICC (95% CI)	Retest ICC (95% CI)	Test ICC (95% CI)	Retest ICC (95% CI)
Knee Right	0.99 (0.97–0.99)	0.98 (0.97–0.99)	0.98 (0.95–0.99)	0.97 (0.95–0.99)
Knee Left	0.98 (0.97–0.99)	0.99 (0.97–0.99)	0.88 (0.79–0.94)	0.97 (0.95–0.99)
Hip Right	0.96 (0.93–0.98)	0.99 (0.97–0.99)	0.94 (0.88–0.97)	0.97 (0.95–0.99)
Hip Left	0.97 (0.94–0.98)	0.98 (0.97–0.99)	0.93 (0.87–0.97)	0.97 (0.94–0.98)

ICC: Intra-class correlation coefficient (mean measure, two-way mixed-effects model where people effects are random and measures effects are fixed, absolute agreement, higher than 0.90 was considered excellent reliability), CI: Confidence interval.

**Table 2:** Comparisons of differences between Kinovea and D-wall.

		Kinovea–D-Wall				
		Correlation (95% CI)	ICC (95% CI)	SEM	Mean Difference (95% CI)	Paired *t* Test *p*-Value *
First evaluation (test)	Knee Right	0.88 (0.72–0.95)	0.94 (0.87–0.97)	4.36	2.16 (−0.19–4.50)	0.070
	Knee Left	0.86 (0.69–0.94)	0.88 (0.76–0.94)	6.54	0.06 (−3.46–3.58)	0.972
	Hip Right	0.92 (0.82–0.97)	0.92 (0.84–0.96)	3.79	1.53 (−0.51–3.57)	0.135
	Hip Left	0.93 (0.83–0.97)	0.93 (0.85–0.97)	3.65	1.71 (−0.25–3.68)	0.085
Second evaluation (retest)	Knee Right	0.99 (0.97–1.00)	0.87 (0.75–0.94)	5.10	−1.44 (−4.19–1.30)	0.290
	Knee Left	0.83 (0.64–0.93)	0.88 (0.76–0.94)	5.01	−1.87 (−4.57–0.82)	0.166
	Hip Right	0.93 (0.84–0.97)	0.94 (0.87–0.97)	3.92	−0.21 (−2.32–1.90)	0.841
	Hip Left	0.93 (0.83–0.97)	0.94 (0.88–0.97)	3.76	−0.18 (−2.20–1.85)	0.860

ICC: Intra-class correlation coefficient (ICC average measure: two-way mixed-effects model where people effects are random and measure effects are fixed; a consistency agreement higher than 0.76 was considered good and 0.90 denoted excellent reliability). CI—Confidence interval. SEM—Standard error of measurement. * There was no statistically significant difference between the Kinovea and D-wall measurements (Paired *t* test *p* values > 0.05). The obtained correlation coefficient was significant (*p* < 0.05).

**Table 3:** Test-retest reliability values of Kinovea and D-Wall.

		Test–Retest			
		ICC (95% CI)	SEM	Mean Diffirence (95% CI)	Paired *t* Test *p*-Value *
Kinovea	Knee Right	0.93 (0.84–0.97)	6.06	−0.53 (−3.79–2.73)	0.743
	Knee Left	0.92 (0.84–0.96)	5.93	−1.11 (−4.30–2.08)	0.481
	Hip Right	0.89 (0.76–0.95)	6.75	1.8 (−1.83–5.43)	0.319
	Hip Left	0.88 (0.75–0.94)	6.89	2.36 (−1.35–6.07)	0.203
D-wall	Knee Right	0.83 (0.63–0.92)	8.25	−4.13 (−8.57–0.31)	0.067
	Knee Left	0.79 (0.55–0.90)	10.51	−3.05 (−8.7–2.61)	0.279
	Hip Right	0.80 (0.57–0.91)	8.48	0.06 (−4.51–4.62)	0.980
	Hip Left	0.79 (0.56–0.9)	8.50	0.47 (−4.1–5.04)	0.834

ICC—Intra-class correlation coefficient (ICC average measure: a two-way mixed-effects model where people effects are random and measure effects are fixed; an absolute agreement higher than 0.76 was considered good and 0.90 denoted excellent reliability). CI—Confidence interval. SEM—Standard error of measurement. * There was no statistically significant difference between the test and re-test measurements (Paired *t* test *p* values > 0.05).

## Data Availability

The data supporting the findings of this study are available from the corresponding author upon reasonable request. While the key results and analyses are included in this manuscript, raw data is not directly presented but can be accessed as needed for further verification or exploration.
